# Machine Learning Prediction Model to Predict Length of Stay of Patients Undergoing Hip or Knee Arthroplasties: Results from a High-Volume Single-Center Multivariate Analysis

**DOI:** 10.3390/jcm13175180

**Published:** 2024-08-31

**Authors:** Vincenzo Di Matteo, Tobia Tommasini, Pierandrea Morandini, Victor Savevski, Guido Grappiolo, Mattia Loppini

**Affiliations:** 1 Department of Biomedical Sciences, Humanitas University, Pieve Emanuele, 20090 Milan, Italy; drvincenzodimatteo@gmail.com; 2 Orthopedics and Trauma Surgery Unit, Department of Aging, Orthopedic and Rheumatologic Sciences, Fondazione Policlinico Universitario Agostino Gemelli IRCCS, 00168 Rome, Italy; 3 IRCCS Humanitas Research Hospital, Rozzano, 20089 Milan, Italy; guido.grappiolo@me.com; 4 Artificial Intelligence Center, IRCCS Humanitas Research Hospital, Via Manzoni 56, Rozzano, 20089 Milan, Italy; tobiatommasini@gmail.com (T.T.); pierandrea.morandini@humanitas.it (P.M.); victor.savevski@humanitas.it (V.S.); 5 Fondazione Livio Sciutto Onlus, Campus Savona, Università Degli Studi di Genova, 17100 Savona, Italy

**Keywords:** artificial intelligence, machine learning, arthroplasty, hip, knee, length of stay

## Abstract

**Background:** The growth of arthroplasty procedures requires innovative strategies to reduce inpatients’ hospital length of stay (LOS). This study aims to develop a machine learning prediction model that may aid in predicting LOS after hip or knee arthroplasties. **Methods:** A collection of all the clinical notes of patients who underwent elective primary or revision arthroplasty from 1 January 2019 to 31 December 2019 was performed. The hospitalization was classified as “short LOS” if it was less than or equal to 6 days and “long LOS” if it was greater than 7 days. Clinical data from pre-operative laboratory analysis, vital parameters, and demographic characteristics of patients were screened. Final data were used to train a logistic regression model with the aim of predicting short or long LOS. **Results:** The final dataset was composed of 1517 patients (795 “long LOS”, 722 “short LOS”, *p* = 0.3196) with a total of 1541 hospital admissions (729 “long LOS”, 812 “short LOS”, *p* < 0.001). The complete model had a prediction efficacy of 78.99% (AUC 0.7899). **Conclusions:** Machine learning may facilitate day-by-day clinical practice determination of which patients are suitable for a shorter LOS and which for a longer LOS, in which a cautious approach could be recommended.

## 1. Introduction

Total hip and knee arthroplasty (THA and TKA) procedures are growing in numbers worldwide each year, with proven improvements in patients’ quality of life [1]. In the USA, as the population progressively ages, the demand for these procedures is expected to grow by 174% for primary THAs and 673% for primary TKAs by 2030. The Italian Arthroplasty Register reported 29,681 THA procedures (94.7% were primary THA and 84.6% were elective procedures) and 19,402 TKA procedures (94.6% were primary TKA) during 2020 [2]. The number of THA and TKA procedures has increased on average by 4.2% each year since 2001 [3]. The rising number of hip and knee arthroplasties has allowed the development of advanced and less invasive surgical techniques, the improvement of perioperative course in order to achieve the shortest average length of stay (LOS) for hospitalization, and a quicker resumption of daily activities, maintaining a low number of complications. Thus, an emerging interest in “fast-track” postoperative protocols erupted over the last several years [4,5]. Frassanito et al. highlighted the impact of the implementation of the enhanced recovery after surgery (ERAS) program for hip and knee replacement procedures, which allowed patients’ early discharge and quick return to independence in daily activities [6]. Despite the enormous increase of the procedures performed, reimbursement for THA and TKA has been dropping throughout recent years, considering that they are not following the trend of the inflation worldwide [7]. The reduction of the reimbursement is in part justifiable by the relatively lower complexity of the younger patients undergoing arthroplasty. On the other hand, whilst the number of patients is increasing, a parallel increase in the complications related to this procedure cannot be accepted. Thus, a public health strategy aimed to reduce costs in economic, social, and health terms is mandatory.

Machine learning has become increasingly applied to medicine and to the orthopedic field, as it represents a natural extension of traditional statistical approaches [8]. Clinical decision support tools that use machine learning algorithms such as random forests, artificial neural networks, or support vector machines have been proven useful in medical research [9,10]. They have the potential to forecast the episode of care by predicting payment or LOS for any given patient after THA and TKA prior to the initiation of the elective procedures [11]. Navarro et al. showed that LOS and cost could be predicted before TKA by a machine learning model using the New York State administrative database [12]. Random forest (RF), an intricate tree-based machine learning algorithm, was used to predict LOS after shoulder arthroplasty [13]. Bayesian algorithms that use conditional probabilities were used to predict LOS and costs after TKA. Etzel et al. used six different machine learning classification algorithms to predict long LOS of anterior and posterior lumbar fusion patients [14]. In recent years, only a few projects have investigated how to facilitate ERAS protocols in the orthopedic field [13,15]; however, machine learning algorithms could be regularly used in clinical practice, employing their potential utility to integrate computerized models into electronic health record systems, where they can be used as point-of-care decision support tools for surgeons. Although a few studies have already investigated the application of machine learning algorithms predicting LOS in patients that received THA and TKA, they were national studies and have all used large administrative datasets. To the best of our knowledge, all these studies investigated patients who underwent only primary THA and TKA [12,16,17,18,19]. Previous studies showed that a small amount of recent and accurate data is more effective than using larger amounts of older data [19]. Therefore, further independent single-center cohort studies are required to confirm these findings.

The purpose of this study was to develop and validate a machine learning algorithm-based prediction tool of pre-operative patient-specific objective criteria, perform multivariable analysis to predict LOS after primary and revision THA or TKA, and elucidate factors correlated with an extended LOS in a high-volume single center. Our hypothesis was that the presented tool can firmly distinguish patients with a predicted “short LOS” if they had LOS less than or equal to 6 (5th postoperative day) and “long LOS” if they had LOS greater than 7 (6th postoperative day), thus giving an advantage in the health management strategies of patients undergoing arthroplasty.

## 2. Materials and Methods

The study was conducted in accordance with the Declaration of Helsinki and good clinical practice guidelines. The study protocol for the development of this registry was approved by the Ethics Committee (protocol code 83/23) of Humanitas Research Hospital IRCCS on July 2023.

### 2.1. Dataset

**Figure 1:**
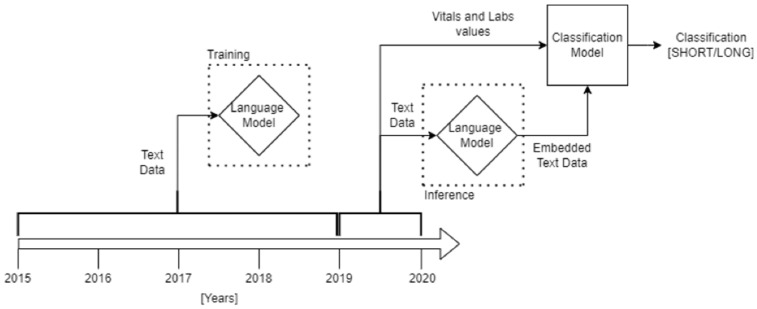
Project setup, how data were used to create the Language Model and to feed the final Classification Model.

Patient-specific data written in medical records from 2015 to 2019 at the Humanitas Research Hospital were used. Textual data from 1 January 2015 to 31 December 2018 were gathered from the collection of all the clinical notes regarding medical history, comorbidities, disabilities, reason for admission, and lower-limb physical examinations. These training set data were used to develop and train an embedding model. Secondly, clinical and textual data from 1 January 2019 to 31 December 2019 coming from pre-operative laboratory analysis, vital parameters, demographics, and morphological characteristics of the selected cohort of patients were screened and used to develop and train a logistic regression machine learning model predicting LOS (Figure 1). Thus, the two sources of data have been merged to create a unique dataset.

### 2.2. Data Extraction

The first data extraction step consisted in querying the data from Data Warehouse (DWH). Oracle SQL^TM^ has been used to gather the relevant data of patients admitted at the orthopedics department. Consequently, a pre-process pipeline has been implemented to clean the text data of unwanted or unnecessary characters, returning a cleaned corpus ready to be processed. The pre-processing phase aimed to normalize the character to ASCII format and remove all present HTML special characters.

### 2.3. Data Selection and Inclusion Criteria

The study included patients undergoing elective primary and revision THA or TKA by senior surgeons experienced in joint replacement surgery, from 1 January 2019 to 31 December 2019, at Humanitas Research Hospital, Italy. Patients were identified from hospital clinical records using International Classification of Diseases, Ninth Revision, Clinical Modification (ICD9-CM) procedure codes (81.51 for THA; 00.70, 00.71, 00.72, 00.73 for revision THA; 81.54 for TKA; 80.06, 81.55, 00.80, 00.81, 00.82, 00.83, 00.84 for revision TKA). Eligibility criteria included all patients aged above 18 years old who underwent elective primary and revision THA or TKA in our orthopedic department. Malunion or nonunion sequelae, traumatology surgeries, not having undergone elective procedures, and malignancy in which LOS could be potentially prolonged in order to achieve histological or cultures assay before patient discharge were grounds for exclusion. Patients who did not have at least 70% of the required predictive features recorded were excluded. Since the management of the postoperative hospitalization and admissions to the rehabilitation unit varied significantly from 2015 to 2019, and since these variations were exogenous, all patients admitted before 2019 in the orthopedic department who underwent primary and revision THA or TKA were further excluded. LOS corresponded to the number of inpatient days during admission: it included the day of patient’s admission which always corresponds to the day before surgery, the day of surgery, and the related postoperative days. Total LOS associated with each patient has been transformed to a categorical feature according to the following decision rule: LOS 0 corresponded to the day of hospital admission, LOS 1 corresponded to the day of surgery, LOS 2 corresponded to the 1st postoperative day, LOS 3 corresponded to the 2nd postoperative day, LOS 4 corresponded to the 3rd postoperative day, LOS 5 corresponded to the 4th postoperative day, LOS 6 corresponded to the 5th postoperative day, LOS 7 corresponded to the 6th postoperative day, and so on. Patients were labelled as ”short LOS” if they had LOS less than or equal to 6 (5th postoperative day), and “long LOS” if they had LOS greater than 7 (6th postoperative day). Patients with LOS equal to 7 (6th postoperative day) were excluded. Finally, a some predictive features selection was required. The first step was to eliminate all variables with a quote of missing values above or equal to 25%. The second step consisted in selecting the relevant clinical features (labs and vital parameters). Then a *p*-value test was used to look for significant differences in predictive feature distributions between “long LOS” and “short LOS” classes and select the ones with a *p*-value lower than 0.05.

### 2.4. Methods

A total of 22 independent variables were collected for each patient and were used for modeling analysis in this study. The patient-related characteristic included age, gender, BMI, marital status (whether the patient was living alone or with family), height, weight, absolute eosinophils count, alanine aminotransferase, anisocytosis index, aspartate aminotransferase, creatinin, erythrocytes, ferritin, hematocrit, hemoglobin, INR, iron, RBC hemoglobin concentration, bilirubin, joint involved, and primary or revision arthroplasty performed. Since the outcome of the study was binary, the problem could be treated as a standard binary classification and be solved using standard supervised learning techniques. In this case, data came from mixed sources, and a certain level of feature engineering was required. In particular, textual data needed to be transformed to numerical vectors to be used in machine learning models (embedding). For this reason, a custom neural network architecture was built. This architecture was able to transform text data while maintaining their local and global structure. This step allowed us to bring data to a common structure and join all different sources of data into one unique dataset. After the embedding procedure, the final data were used to train a logistic regression model to predict whether a patient was more likely to have a long or short LOS following primary or revision THA and TKA. All procedures described above have been performed using Python 3.9. In particular, the following libraries have been used:

- Pandas 1.0.1 [20]: importing and managing data.
- Numpy 1.18.1 [21]: array manipulation and scientific computation.
- Scikit-learn 1.0.0 [22]: definition, training, and validation of machine learning and statistical models.
- Tensorflow 2.0.0 [23]: definition, training, and validation of transformer autoencoder.
- Matplotlib 3.1.3 [24]: plotting models’ performances.
- Scipy 1.5.2 [25] and Statsmodels 0.12.0 [26]: performing statistical tests.

### 2.5. Text Pre-Processing

**Figure 2:**
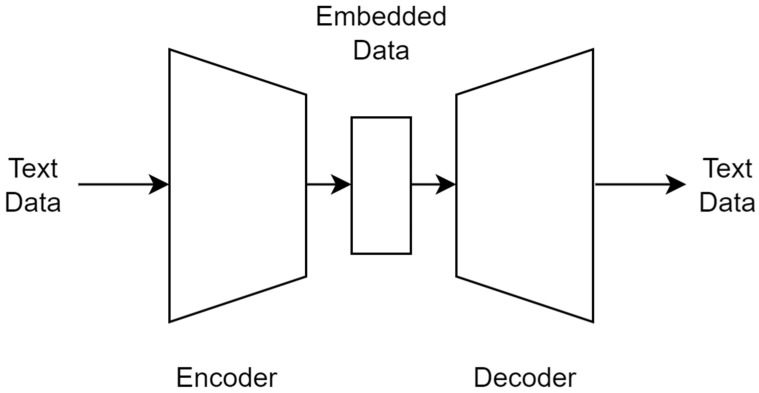
The autoencoder structure was composed by an encoder and a decoder. The encoder compressed the input information, usually unstructured information like images or texts, in a numeric format producing the embedding. The decoder took as input the embedded data and tried to reconstruct the data in its original format. During the training process, the two parts cooperated to compress and reconstruct the input data as accurately as possible. In this project, after the training, only the encoder was used during the final classification to encode the data in numeric format to feed the logistic regression performing the classification.

A pre-trained deep learning architecture able to extract relevant information from clinical texts was not available. The majority of pre-trained language models based on deep learning algorithms were trained on generic corpuses [27] and could not be used on specific texts like the ones here considered, because this would likely result in poor latent representation. Therefore, a custom neural network architecture to encode data coming from anamnesis, previous surgeries, and reason for admission into 300-dimensional numerical vectors was developed. From an architectural point of view, the network was designed as a transformer autoencoder, with both encoder and decoder composed of 3 three-headed attention layers (Figure 2) [28].

The model was trained by the Adaptive Moment Estimation (ADAM) [29] algorithm using all the clinical notes regarding anamnesis, comorbidities, disabilities, reasons for admission, and lower-limb clinical examinations, written from 2015 to 2018, with a train and validation set consisting of, respectively, 36,489 and 9153 clinical sentences, with the aim of minimizing a loss of function based on binary cross-entropy. At the end of the process, the encoded texts were reduced, using principal component analysis, to a dimensionality able to explain 90% of the variance, resulting in 48-dimensional vectors for anamnesis, 58-dimensional for previous surgeries, and 16-dimensional for reason for admission.

### 2.6. Classification

The sources of data for the analysis were different: 79.2% of features came from textual data, while 20.8% came from labs and morphological and demographic features. The study was structured in order to understand the impact of all the different sources, defining three different models using three different sets of features: the first which used only laboratory exams and demography features (structured data), the second which used only text-derived features (unstructured data), and a third which used both structured and unstructured data. Subsequently, models’ performances were compared using standard classification scores and AUC. From an architectural point of view, all models were structured as three-layered pipelines with a z-score-based standardizer as first layer, an iterative imputer based on chained equations [30] to impute missing values as second layer, and a logistic regression classifier as last layer. Hyperparameters for all models were chosen using a randomized search algorithm, and the training and testing procedure were performed using the hold-out strategy, in which data were randomly split according to the following decision rule: 70% for the training phase and 30% for the testing one.

### 2.7. Statistical Analysis

The statistical analysis was mainly focused on understanding the impact of the selected covariates on the outcome distribution. Since text-embedding vectors were built using deep learning, all the interpretability was lost in the process, and the inference part could only be done on laboratory exams, demographic data, and morphological features. With respect to univariate analysis, the distribution was divided according to LOS, as mentioned before, and a Mann–Whitney U test [31] or *t*-test [32] was used for continuous variables according to the result of the Shapiro–Wilk test [33] used to assess normality. For categorical features, a proportion Z-test (or two classes of Chi-squared [34]) was used, with the aim of assessing significant differences in the features distribution. A multivariate analysis was performed using logistic regression [35], to compute risk factors (odds ratios) and their relative confidence intervals. To assess the significance of the odds ratio, a *t*-test was performed and *p*-values of the Wald statistics were obtained. Finally, all *p*-values below 0.05 were considered as statistically significant.

## 3. Results

### 3.1. Dataset and Univariate Analysis

The final dataset was composed of 1517 extracted and identified patients, of which 795 belonged to class “long LOS” and 722 to class “short LOS” (*p* = 0.3196), with a total of 1541 admissions, 729 (47.3%) belonging to Group 1, “short LOS”, and 812 (52.7%) belonging to Group 2, “long LOS” (*p* < 0.001). Average LOS was 11.7 and 5.7 for “long LOS” and “short LOS”, respectively. Group 1 included 729 patients with a mean age of 63.8 (20–90; σ 12.1) years old. There were 364 (49.9%) female patients and 365 (50.1%) male patients; 36 (4.9%) patients underwent bilateral arthroplasty, 722 (99.0%) underwent primary arthroplasty, and 7 (1.0%) underwent revision arthroplasty. Group 2 included 812 patients with a mean age of 70.0 (14–90; σ 12.1) years old. There were 503 (61.9%) female patients (*p* < 0.001) and 309 (38.1%) male patients (*p* < 0.0023); 641 (78.9%) underwent primary arthroplasty and 171 (21.1%) underwent revision arthroplasty. In Group 1, 174 (21.4%) patients underwent bilateral arthroplasty, 530 (72.7%) patients underwent hip arthroplasty, and 199 (27.3%) patients underwent knee arthroplasty. In Group 2, 639 (78.7%) patients underwent hip arthroplasty (*p* < 0.001) and 173 (21.3%) patients underwent knee arthroplasty (*p* < 0.057). Among 178 revision arthroplasties, 7 (3.9%) were performed in Group 1 and 171 (96.1%) were performed in Group 2 (*p* < 0.001). Among 1363 primary arthroplasties, 722 (53.0%) were performed in Group 1 and 641(47.0%) were performed in Group 2 (*p* = 0.002). Finally, 565 admissions needed to be moved to the rehabilitation unit, 1 (0.2%) in Group 1 and 564 (98.8%) in Group 2 (*p* < 0.001); 976 patients did not, 728 (74.6%) in Group 1 and 248 (25.4%) in Group 2 (*p* < 0.001). Demographic, clinical, and morphological features are reported in Table 1.

### 3.2. Classification

All the features used in the model were information available at pre-admission level. This includes gender, age, BMI, height, weight, body part, marital status, and revision flag (a flag indicating whether a surgery is a revision or a primary arthroplasty). In addition to this, all laboratory analysis described in Table 1 was included. The complete model including all sources of features was the best-performing one, with an area under the curve (AUC: 0.7899), followed by texts model (AUC: 0.7228) and labs and demos model (AUC: 0.7198). Apart from AUC, the dominance of the complete model was confirmed by all the selected classification scores (Table 2 and Table 3) as well as the AUC order.

### 3.3. Multivariate Analysis

**Figure 3:**
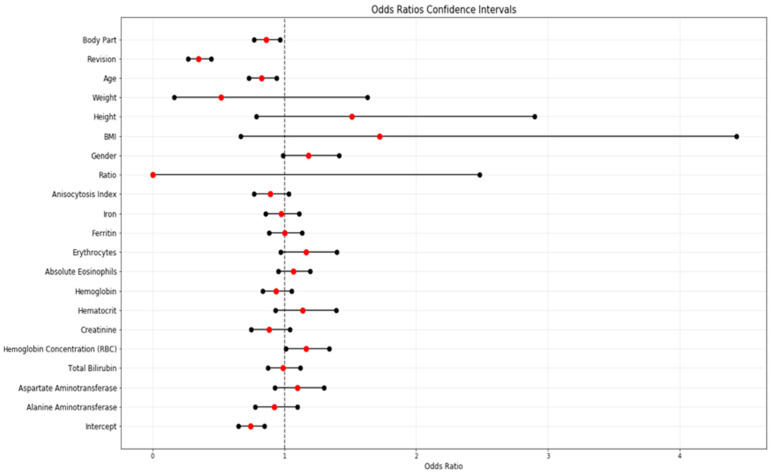
Multivariate logistic regression odds ratios and CIs. (Note: INR has been removed from the plot due to a scale problem).

Multivariate logistic regression results can be found in Table 4 and Figure 3. The model was fitted using the BFGS algorithm and without adding any regularization term. This allows us to obtain unbiased estimators of the LR coefficients [36].

## 4. Discussion

**Figure 4:**
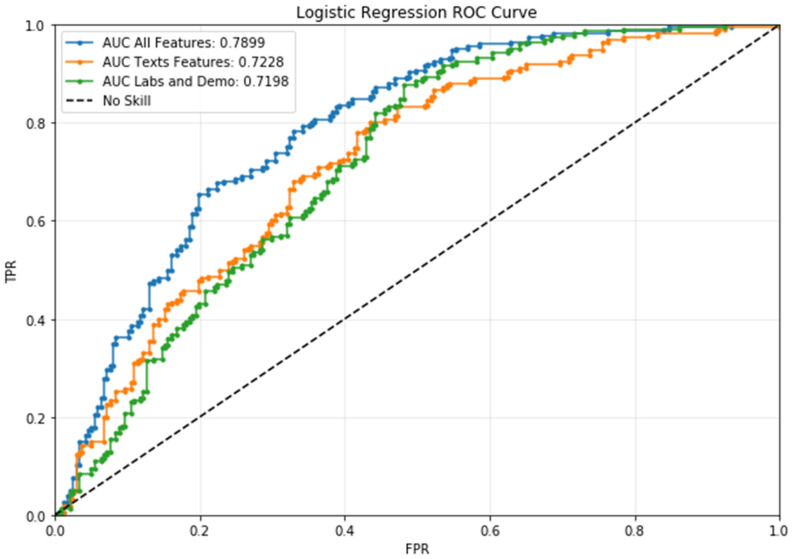
This confirms the results shown in Table 2 and Table 3: the ROC curve for the complete model dominates the others.

The main finding of our study was the capability of the machine learning algorithm in predicting LOS in patients undergoing elective primary or revision THA or TKA. This tool could forecast patients as candidates for “short LOS” if they had LOS less than or equal to 6 days and “long LOS” if they had LOS greater than 7 days with great accuracy, taking into consideration data extracted from pre-admission routine. Thus, patients suitable for ERAS protocols could be identified at pre-admission. From a methodological point of view, the most interesting result of the study was the comparison between different sources of data. As a matter of fact, it was possible to understand the information added by each subset of considered features and by their combination (Table 2 and Table 3). As previously stated, it is straightforward to see the dominance of the complete model, which relies on information provided by clinical texts, labs, demographics, and morphological features. Secondly, it is also interesting to focus on the performances of the two control models: Table 2 and Table 3 and Figure 4 show similar results, especially for the AUC. Moreover, ROC curves intersect at about 0.8, 0.42, making these more difficult to interpret. Overall, the results reported here showed that much clinical information could be extracted from texts, and this relates to physicians’ experience and the quality of physical examination performed. On the other hand, the significant boost in all classification performances given by adding labs and demographic and morphological features to the model indicates that documents written by clinicians were not able to capture all the information needed to perform a correct classification. Taking into account the clinical relevance of the present study, the evaluation of comorbidities is very important given the type of patients undergoing arthroplasty, especially if an ERAS protocol is advocated. When Moldovan investigated bone cement implantation syndrome (BCIS), a sporadic and potentially lethal complication after THA, in our high-volume single center for prosthetic surgery, no BCIS occurred and no significant difference in LOS after cemented and uncemented hip arthroplasty was recorded, and therefore stem cementation was not considered as a relevant feature [37].

Previous studies used machine learning to predict LOS after TKA, THA, and TSA with c-statistics of 0.78, 0.87, and 0.77, respectively. The present model had the potential to be integrated into the electronic medical record to provide a personalized assessment of a patient’s potential need for a longer or shorter LOS in the hospital after undergoing total joint arthroplasty, hospital readmission, or reintervention [12,13,17]. Podmore et al. [38] in 2021 included 640,832 patients who had a primary hip or knee arthroplasty between April 2009 and March 2016 in a study evaluating the impact of 11 comorbidities on the safety risks (including LOS and 30-day readmission rate) of hip and knee arthroplasty surgery. The present model included all the comorbidities. Their study highlighted the impact of the examined comorbidities on clinical and socioeconomical fields. Alternatively, they concluded that the increased risk is small compared with the large improvements in functional outcomes, even in patients with multiple comorbidities. Thus, a prediction based on pre-admission evaluation of comorbidities and labs could help to individualize the path of the patients from admission to complete recovery. Zhu et al. in 2017 [6] performed a large meta-analysis of RCTs and CCTs available on literature about ERAS protocols in arthroplasty surgery. They concluded that ERAS significantly reduces LOS and incidence of complications in patients who have undergone THA or TKA. One of the most interesting aspects that emerged throughout their study was the need for improvement of perioperative management of the patient over the surgical technique. In this scenario, the correct selection of patients eligible for the ERAS protocol was the crucial aspect to enhance clinical outcomes. Furthermore, ERAS protocols have shown positive effects in early rehabilitation: Masaracchio et al. in 2017 [39] summarized the beneficial effects of an early administration of rehab protocols. Early rehabilitation reduced LOS and socioeconomical cost of the procedure. Despite these beneficial effects, early mobilization could lead to complications like falls if addressed to patients with a certain risk profile (i.e., cardiovascular or neurological disease). To avoid such complications, a quantitative, individualized risk assessment through artificial intelligence could be beneficial. Focusing on the economical aspect, reimbursement for THA and TKA dramatically dropped over the last 20 years, especially considering inflation [7]. The amount of reimbursement is strictly linked with patient volume, patient satisfaction, a healthier patient population, and government ownership of a hospital, as stated by Padegimas et al. in 2016 [40]. A predictive tool for the enhancement of selection of patients eligible for ERAS protocol could help in the path towards a more sustainable arthroplasty surgery in the context of limited resources. A similar machine learning approach was evaluated by Anis et al. in 2020 [9]. Their study was focused on predicting LOS using a Poisson regression model. One similarity with our approach was the feature selection process; in fact, they chose to focus on laboratory analysis as well as patients’ anamnestic details. However, their study was prospective, and features were specifically selected for the task. Such features were demographics and specific clinical scores from previous examinations. Our study, on the other hand, was retrospective, and the main feature selection process was more general and focused on features routinely collected during daily examination activities. Considering that we did not have direct access to all the specific clinical information, we used the transformer architecture to automatically extract proxies of this information from the selected texts. A rigorous comparison of the two studies cannot be assessed since they are based on different modeling strategy. However, our approach may have a significant boost in simplicity of data collection, and thus can be more easily implemented as a routine clinical service since, as previously stated, our features can be easily retrieved from hospital daily practice.

## 5. Limitations of the Study and Future Plans

This study had several potential limitations. First, it relied on textual data, and information coming from clinical records needed to be heavily pre-processed to be used by language models, resulting in a more difficult prediction process. Second, some interpretability was lost in the embedding process: the vectorization of the documents made causality between the presence of a token (a word or a sentence) and the selected outcomes difficult to assess, making data more suitable for prediction tasks. Third, our language model was trained and validated only on internal data, and an external validation was required as a benchmark. Fourth, all the clinical texts were written in Italian, so the model is well-suited only for one language.

Future research activities will involve collaborating with external hospitals to create a larger and more diverse cohort of patients. This will help confirm the reported findings and develop more inclusive models, reducing potential biases that may be inherent in the data collection process. Incorporating external data will also enhance the text embedding model, which, in this instance, was trained only on data from a single facility. Including text data from different institutions could improve the model’s understanding, leading to a better representation of the clinical state of patients.

## 6. Conclusions

This study demonstrated the reliability of an artificial intelligence model to distinguish fit patients suitable for a shorter LOS, thus eligible for ERAS protocols, and patients with an expected longer LOS. The promising results suggest the potential utility of integrating computerized algorithms in electronic health record systems, where they can be used as a point-of-care decision support tool to assist the surgeons in patient selection. As these decision support tools become part of regular practice, however, they should not replace the clinical judgment of the surgeon, but rather supplement the informed consent process and contribute to shared decision making. Further, prospective studies are needed to validate our findings and the feasibility of this technology in clinical practice.

## Figures and Tables

**Table 1:** Demographic, clinical, and morphological characteristics of the cohort.

		Long	Short	*p*-Value
Patients, *n*		795	722	
Admissions, *n*		812	729	
Age, mean (SD)		67.0 (12.9)	63.8 (10.9)	<0.001
BMI, mean (SD)		27.4 (5.0)	27.7 (4.5)	0.260
Height, mean (SD)		165.7 (9.8)	167.7 (9.2)	<0.001
Weight, mean (SD)		75.6 (16.3)	78.2 (15.3)	0.001
Gender, *n* (%)	Female	503 (61.9)	364 (49.9)	<0.001
	Male	309 (38.1)	365 (50.1)	
Alanine aminotransferase, mean (SD)		19.2 (11.1)	21.2 (12.3)	0.001
Aspartate aminotransferase, mean (SD)		21.4 (7.5)	22.3 (10.8)	0.041
Total bilirubin, mean (SD)		0.7 (0.3)	0.7 (0.3)	0.030
RBC hemoglobin concentration, mean (SD)		33.1 (0.8)	33.3 (0.8)	<0.001
Creatinin, mean (SD)		0.8 (0.4)	0.8 (0.2)	<0.001
Hematocrit, mean (SD)		41.8 (3.8)	43.2 (3.7)	<0.001
Hemoglobin, mean (SD)		7.5 (2.0)	7.7 (2.0)	<0.001
Absolute eosinophils, mean (SD)		0.2 (0.1)	0.2 (0.1)	0.044
Erythrocytes, mean (SD)		4.6 (0.5)	4.8 (0.4)	<0.001
Ferritin, mean (SD)		96.2 (89.3)	108.1 (89.6)	0.009
Iron, mean (SD)		80.9 (30.1)	86.5 (29.8)	<0.001
INR, mean (SD)		1.1 (0.2)	1.0 (0.1)	<0.001
Anisocytosis index, mean (SD)		14.3 (1.3)	14.0 (1.2)	<0.001
Ratio, mean (SD)		1.1 (0.2)	1.0 (0.1)	<0.001
Revision, *n* (%)	No	641 (78.9)	722 (99.0)	<0.001
	Yes	171 (21.1)	7 (1.0)	
Body part, *n* (%)	Hip	639 (78.7)	530 (72.7)	0.007
	Knee	173 (21.3)	199 (27.3)	
Marital status, *n* (%)	Five	11 (1.4)	3 (0.4)	0.283
	Four	2 (0.2)	1 (0.1)	
	One	6 (0.7)	4 (0.5)	
	Six	560 (69.0)	481 (66.0)	
	Three	2 (0.2)	1 (0.1)	
	Two	60 (7.4)	59 (8.1)	
	Unknown	171 (21.1)	180 (24.7)	

BMI, body mass index; INR, international normalized ratio; RBC, red blood cell.

**Table 2:** Model classification scores for the two classes.

	Long				Short			
	F1 Score	Precision	Recall	Support	F1 Score	Precision	Recall	Support
Complete	0.709251	0.741935	0.679325	237.0	0.720339	0.691057	0.752212	226.0
Texts	0.656319	0.691589	0.624473	237.0	0.673684	0.642570	0.707965	226.0
Others	0.642082	0.660714	0.624473	237.0	0.645161	0.627615	0.663717	226.0

**Table 3:** Model classification scores (averages).

	Macro Avg.				Weighted Avg.			
	F1 Score	Precision	Recall	Support	F1 Score	Precision	Recall	Support
Complete	0.714795	0.716496	0.715769	463.0	0.714663	0.717101	0.714903	463.0
Texts	0.665002	0.66708	0.666219	463.0	0.664795	0.667662	0.665227	463.0
Others	0.643622	0.644165	0.644095	463.0	0.643585	0.644558	0.643629	463.0

**Table 4:** Multivariate logistic regression results. (Note: missing values correspond to negative variance estimation).

	Feature	Coefficients	Standard Errors	W Values	*p* > |z|	Odds Ratio	[0.025]	[0.975]
0	Intercept	−0.2990	0.0680	−4.3950	<0.0001	0.7416	0.6490	0.8473
1	Alanine aminotransferase	−0.0785	0.0870	−0.9060	0.3650	0.9245	0.7796	1.0964
2	Aspartate aminotransferase	0.0931	0.0860	1.0860	0.2780	1.0976	0.9273	1.2991
3	Total bilirubin	−0.0111	0.0630	−0.1760	0.8600	0.9890	0.8741	1.1189
4	Mean corpuscular hemoglobin concentration (MCHC)	0.1503	0.0720	2.0910	0.0370	1.1622	1.0092	1.3383
5	RBC hemoglobin concentration	−0.1261	0.0850	−1.4880	0.1370	0.8815	0.7462	1.0413
6	Hematocrit	0.1303	0.1020	1.2820	0.2000	1.1392	0.9327	1.3913
7	Hemoglobin	−0.0647	0.0600	−1.0800	0.2800	0.9373	0.8334	1.0543
8	Absolute eosinophils	0.0658	0.0570	1.1600	0.2460	1.0680	0.9551	1.1943
9	Erythrocytes	0.1528	0.0930	1.6510	0.0990	1.1651	0.9710	1,3981
10	Ferritin	0.0012	0.0630	0.0190	0.9850	1.0012	0.8849	1.1328
11	Iron	−0.0239	0.0670	−0.3560	0.7220	0.9764	0.8562	1.1134
12	INR	8.4158	4.9490	1.7000	0.0890	4517.8884	0.2769	73,724,077.5095
13	Anisocytosis index	−0.1163	0.0760	−1.5400	0.1240	0.8902	0.7670	1.0332
14	Ratio	−8.8155	4.9610	−1.7770	0.0760	0.0001	0.0000	2.4795
15	Gender	0.1667	0.0910	1.8380	0.0660	1.1814	0.9884	1.4121
16	Marital status—One	−0.0423	/	0.0000	1.0000	0.9586	0	/
17	Marital status—Two	0.0191	/	0.0000	1.0000	1.0193	0	/
18	Marital status—Three	−0.0360	/	0.0000	1.0000	0.9646	0	/
19	Marital status—Four	−0.0491	/	0.0000	1.0000	0.9521	0	/
20	Marital status—Five	−0.0973	/	0.0000	1.0000	0.9073	0	/
21	Marital status—Six	0.0011	/	0.0000	1.0000	1.0011	0	/
22	Marital status—Unknown	0.0257	/	0.0000	1.0000	1.0260	0	/
23	BMI	0.5422	0.4830	1.1220	0.2620	1.7198	0.6673	4.4321
24	Height	0.4118	0.3330	1.2380	0.2160	1.5095	0.7859	2.8993
25	Weight	−0.6558	0.5840	−1.1220	0.2620	0.5190	0.1652	1.6304
26	Age	−0.1895	0.0640	−2.9460	0.0030	0.8274	0.7298	0.9379
27	Revision	−1.0611	0.1260	−8.4100	<0.0001	0.3461	0.2703	0.4430
28	Body Part	−0.1499	0.0590	−2.5560	0.0110	0.8608	0.7668	0.9663

## Data Availability

The data supporting the reported results can be found in a repository (Zenodo).
